# Effects of *Lactobacillus Plantarum* and *Lactobacillus Helveticus* on Renal Insulin Signaling, Inflammatory Markers, and Glucose Transporters in High-Fructose-Fed Rats

**DOI:** 10.3390/medicina55050207

**Published:** 2019-05-24

**Authors:** Omer A. Korkmaz, Esra Sumlu, H. Bugra Koca, M. Bilgehan Pektas, Aytac Kocabas, Gokhan Sadi, Fatma Akar

**Affiliations:** 1 Department of Chemistry, Faculty of Science, Yildiz Technical University, Istanbul, 34220, Turkey; omeradilk@gmail.com; 2 Department of Pharmacology, Faculty of Pharmacy, Gazi University, Ankara, 06330, Turkey; esrasmlu@gmail.com; 3 Department of Medical Biochemistry, Faculty of Medicine, Afyonkarahisar Health Sciences University, Afyonkarahisar, 03200 Turkey; hbkoca@yahoo.com; 4 Department of Medical Pharmacology, Faculty of Medicine, Afyonkarahisar Health Sciences University, Afyonkarahisar, 03200 Turkey; mbpektas@gmail.com; 5 Department of Biology, K.Ö. Science Faculty, Karamanoglu Mehmetbey University, Karaman, 70000 Turkey; aytackocabas@kmu.edu.tr (A.K.); sadi@kmu.edu.tr (G.S.)

**Keywords:** dietary fructose, *Lactobacillus plantarum*, *Lactobacillus helveticus*, insulin signaling, inflammation, glucose transporters, kidney

## Abstract

*Background and Objectives:* The excess consumption of fructose in the diet may cause metabolic syndrome, which is associated with an increased risk of kidney disease. There is limited data on probiotic treatment in high-fructose-induced metabolic syndrome. The present study aims to investigate whether the supplementation of *Lactobacillus* *plantarum (L. plantarum)* and *Lactobacillus* *helveticus* (*L. helveticus)* could provide an improving effect on the renal insulin signaling effectors, inflammatory parameters, and glucose transporters in fructose-fed rats. *Materials and Methods:* The model of metabolic syndrome in male Wistar rats was produced by fructose, which was given as 20% solution in drinking water for 15 weeks. *L. plantarum* and *L. helveticus* supplementations were given by gastric gavage from 10 to 15 weeks of age. *Results:* High-fructose consumption in rats reduced renal protein expressions of insulin receptor substrate (IRS)-1, protein kinase B (AKT), and endothelial nitric oxide synthase (eNOS), which were improved by *L. plantarum* and partially by *L. helveticus* supplementations. Dietary fructose-induced elevations in renal tissue levels of tumor necrosis factor α (TNF-α), interleukin (IL)-1β, IL-6, and IL-10, as well as expression of IL-6 mRNA, were attenuated, especially in *L. plantarum* treated rats. The increased renal expression of sodium-glucose cotransporter-2 (SGLT2), but not that of glucose transporter type-5 (GLUT5), was suppressed by the treatment with *L. plantarum*. *Conclusion:* Suppression in insulin signaling pathway together with the induction of inflammatory markers and upregulation of SGLT2 in fructose-fed rats were improved by *L. plantarum* supplementation. These findings may offer a new approach to the management of renal dysregulation induced by dietary high-fructose.

## 1. Introduction

Type 2 diabetes causes many complications in several organs, including the kidney. Dysfunction in the insulin signaling pathway may cause compensatory hyperinsulinemia, thereby generating resistance to insulin. Insulin resistance, together with inflammatory status, might occur due to excess nutritional fructose and affect the intestinal microbiota [1,2]. In our previous studies, we showed that high-fructose intake changed the expression levels of insulin signaling effectors, such as insulin receptor (IR), insulin receptor substrates (IRS-1 and IRS-2), protein kinase B (AKT), or endothelial nitric oxide synthase (eNOS), as well as activated inflammatory markers in blood vessel, adipose tissue, liver, and testis of rats [3,4,5,6,7,8]. In studies with kidney tissue of high-fructose-fed rats, reduced expression or phosphorylation of IR, IRS-1, and AKT, as well as overproduction of cytokines, such as interleukin (IL)-1β, IL-6, and tumor necrosis factor α (TNF-α), indicated that there is a connection between inflammation and impaired insulin signaling [9,10]. In this line, dietary high-fructose was shown to produce a proinflammatory condition, as evidenced by the activation of nuclear factor-kappa B (NF-κB) and high expression level of TNF-α, inducible NOS (iNOS), and IL-6 in renal tissues of rats [11]. Furthermore, inflammation and the tubulointerstitial injury were associated with an increased expression of glucose transporter type-5 (GLUT5), which is a specific fructose transporter, in renal tissues of rats or mice fed with high-fructose diet [12,13]. On the other hand, renal expression of sodium-glucose cotransporter-2 (SGLT2), which is a major glucose transporter in the proximal tubule, was increased in hyperglycemic conditions, including feeding with high-fructose diet [14].

Probiotics are living microorganisms and widely used to prepare fermented products. The beneficial health effects of probiotics have gained widespread worldwide acceptance; however, new investigations are required to clarify their mechanism of action in specific pathological conditions, such as type 2 diabetes [15,16]. Supplementation with *Lactobacillus (L.)* species, which are one of the primary components of the human intestinal microbiota, has been reported to produce antidiabetic, antihyperlipidemic, anti-inflammatory, and antioxidant effects [17,18,19,20]. Previously, probiotic containing *L. acidophilus* and *L. casei* was reported to restrict hyperglycemia, hyperinsulinemia, and dyslipidemia in high-fructose-fed rats [21]. *L. curvatus* and *L. plantarum* combination regulated the characteristics of metabolic syndrome, and the oxidative stress also reduced hepatic expression of lipogenic genes in the high-fructose-fed rats [22]. Supplementation of *L. plantarum* was shown to improve insulin resistance and capacity of antioxidant enzymes, as well as to attenuate pro-inflammatory cytokines IL-1β, IL-6, and TNF-α in rats fed with high fat-fructose diet [23]. The elevated concentration of IL-6 and TNF-α, as well as decreased expression levels of peroxisome proliferator-activated receptor (PPAR-γ) and GLUT4 mRNAs, in adipose tissue of high-fructose-treated rats, were improved by *L. reuteri* GMNL-263 administration [24]. *L. rhamnosus GG* at a dosage of approximately 5×107 CFU/g body weight was shown to ameliorate fructose-induced liver inflammation and hepatic fatty acid accumulation in mice by decreasing hepatic TNF-α, IL-1β, and IL-8R mRNAs and regulating hepatic fatty acid metabolism, respectively [25]. A limited number of studies, mentioned above, demonstrates the need for mechanistic further studies with probiotics in high-fructose-induced metabolic syndrome. Recently, we have shown that the dietary fructose induced the changes in some metabolic parameters, such as plasma insulin, triglyceride, VLDL, creatinine, and renal urea, as well as in renal protein expression level of superoxide dismutase 1 (SOD1), SOD2, and catalase (CAT), which were improved with supplementation of *L. plantarum* and *L. helveticus* [26]. By extending that study, herein, we assumed that the supplementation of these two probiotics may also affect the renal insulin signaling elements, inflammatory parameters, or glucose transporters in fructose-fed rats.

## 2. Materials and Methods

### 2.1. Animals and Diets

The Ethical Animal Research Committee of Afyon Kocatepe University (Akuhadyek-49533702) on April 25, 2018 approved the protocol for animal usage. Three weeks old male Wistar rats were housed under temperature and humidity-controlled rooms (20–22 °C) with a 12 h light-dark cycle. The animals were fed with a standard rodent chow diet that composed of 62% starch, 23% protein, 4% fat, 7% cellulose, standard vitamins, and salt mixture. At the end of the acclimation for one week, rats were randomly divided into four groups as Control; Fructose (Fruc); Fructose + *L. plantarum* (Fruc + LP), and Fructose + *L. helveticus* (Fruc + LH). Fructose (Danisco Sweeteners OY, Kotka, Finland) was given to the rats as 20% solution (w/v) in drinking water for 15 weeks. *L. plantarum* and *L. helveticus* (Chr. Hansen, Hørsholm, Denmark; ATCC: 14917 and ATCC: 15009, respectively), which are grown in our laboratory, were given to the rats as 1 × 10^+9^ CFU per 100 g of body weight of animal in 2 mL of saline by gastric gavage once a day during final six weeks. The control and fructose groups were administered with the same volume of saline by the gavage for the same period. Body weight, food, and liquid intakes were recorded weekly during the follow-up period. At the end of the follow-up period, the rats were anesthetized with a mixture of ketamine-xylazine (100 and 10 mg/kg, respectively, i.p.). The kidney tissues were blotted dry, frozen in liquid nitrogen, and stored at −85 °C.

### 2.2. Preparation of L. plantarum and L. helveticus 

*L. plantarum* and *L. helveticus* were cultured in De Man, Rogosa and Sharpe broth (MRS; Oxoid; Unipath Ltd., Basingstoke, Hampshire, England) at 30 °C in a rotary shaker at 150 rpm. Stock cultures were stored at −80 °C in MRS broth containing 20% (v/v) glycerol. Erlenmeyer flasks containing 20 mL of MRS were inoculated with 1.5 mL of glycerol stock culture, and the cultures were incubated at 35 °C ± 1 °C in a rotary shaker at 150 rpm and grown to an optical density of 1.0 at 600 nm (cell density corresponding to 1 × 10^8^ CFU/ml). The culture was divided into 10 mL tubes (1 × 10^9^ CFU), and the cells were harvested at 5000 *g* for 5 min at 4 °C. The cell pellets were washed with isotonic saline solution and lyophilized under a freeze dryer.

### 2.3. Measurement of Inflammatory Parameters in Kidney

Kidney samples were homogenized in 0.1 M phosphate buffer (1:10 (w/v), pH 7.4) and 24,000 cycles/min (Ultra Turrax, Ika Works Inc., Wilmington, NC, USA), and then ultrasonicated 20,000 cycles/sec for 1 min (Dr. Hielscher, Teltow, Germany). Homogenates were centrifuged at +4 °C at 10,000× *g* for 15 min, and the supernatants were collected. All samples were stored at −85°C until analysis. Renal tissue levels of NF-κB, TNF-α, IL-1β, IL-6, and IL-10 (eBioscience, San Diego, CA, USA) were measured by using commercial ELISA kits according to the manufacturer’s instructions. 

### 2.4. Determination of Gene Expressions With Quantitative Real-Time Polymerase Chain Reaction (qRT-PCR)

Total RNAs were isolated from the kidney tissues using RNeasy total RNA isolation kit (Qiagen, Venlo, Netherlands), as described according to the manufacturer protocol. After isolation, the amount and the quality of the total RNAs were determined by spectrophotometry and agarose gel electrophoresis. Then, one µg of total RNA was reverse transcribed to cDNA using commercial first strand cDNA synthesis kit (Thermo Fisher Scientific, Waltham, MA, USA). Expression levels of *ir, irs-1, irs-2, akt, enos, nf-κb,* and *il-6* were determined with a real-time quantitative polymerase chain reaction (qRT-PCR, LightCycler480 II, Roche, Basel, Switzerland). To do this, 1 μl cDNA, 5 μl 2X SYBR Green Master mix (Roche FastStart Universal SYBR Green Master Mix, Roche, Basel, Switzerland), and 2 μl primer pairs of each (Table 1) at 0.5 µM concentrations in a final volume of 10 µl were mixed and qRT-PCR was performed as follows: initial denaturation at 95 °C for 10 min, denaturation at 95 °C for 10 s, annealing at 58 °C for 15 s, and extension at 72 °C for 15 s with 40 repeated thermal cycles measuring the green fluorescence at the end of each extension step. All reactions were performed in triplicates, and the specificity of PCR products was confirmed using melt analysis, as we described previously [4,6]. The relative expression of genes with respect to internal control glyceraldehyde 3-phosphate dehydrogenase (*gapdh*) was calculated with the efficiency corrected advance relative quantification tool provided by the LightCycler^®^ 480 SW 1.5.1 software.

### 2.5. Determination of Protein Expressions by Western Blot 

Renal tissues were homogenized in 2-fold volumes of homogenization medium (50 mM Tris, 150 mM sodium chloride, 5 mM ethylenediaminetetraacetic acid, 1% (w/w) Triton X-100, 0.26% (w/v) sodium deoxycholate, 50 mM sodium fluoride, 0.1 mM sodium orthovanadate, and 0.2 mM phenylmethylsulfonyl fluoride, pH: 7.4) with Tissue Ruptor^TM^ (Qiagen, Venlo, Netherlands) homogenizer, and then the homogenates were centrifuged at 1500× *g* for 10 min at +4 °C. After the removal of supernatants, protein concentrations were determined by the Lowry method [27]. A total of 50–100 µg of total proteins from different groups were separated by polyacrylamide gel electrophoresis and transferred onto the polyvinylidene fluoride membranes using a semi-dry electroblotting apparatus (TransBlot Turbo, BioRad, Munich, Germany), after which the membranes were blocked with 5% non-fat dry milk for one hour. Primary antibodies were utilized for priming the respective IRS-1 (anti-IRS-1 rabbit IgG, Santa Cruz, CA, USA, 1:1000), eNOS (anti- eNOS rabbit IgG, Santa Cruz, CA, USA, 1:500), AKT (anti- AKT rabbit IgG, Santa Cruz, CA, USA, 1:1000), SGLT2 (anti-SGLT2 rabbit IgG, BosterBio, Wuhan, China, 1:1000), and GLUT5 (anti-GLUT5 rabbit IgG, BosterBio, Wuhan, China, 1:1000) proteins for 2 h at room temperature or overnight at +4 °C. As an internal control, GAPDH proteins were also labeled with GAPDH (anti-GAPDH Rabbit IgG, Santa Cruz, CA, USA, 1:4000) for the data normalization. After the washing step, horseradish peroxidase (HRP) conjugated secondary antibodies (Goat Anti-rabbit IgG-HRP conjugate; Santa Cruz, CA, USA, 1:10,000) were incubated for 1 h, and then the blots were treated with Clarity^TM^ Western ECL (Bio-Rad Laboratories, Hercules, CA, USA) substrate solution for 5 min. Images of the blots were gained using the ChemiDoc^TM^ MP Chemiluminescence detection system (Bio-Rad Laboratories, Hercules, CA, USA) equipped with a CCD camera. The relative expression of proteins with respect to GAPDH was calculated using the ImageLab 4.1 software (Bio-Rad Laboratories, Hercules, CA, USA).

### 2.6. Measurement of Fructose Level in Plasma and Kidney

Cardiac blood samples of non-fasted rats were immediately centrifuged at 4 °C and 10,000× *g* for 30 min. Kidney samples were homogenized in 0.1 M phosphate buffer 1:10 (w/v), pH 7.4, and 24,000 cycles/min (Ultra Turrax, IKA-Works, Wilmington, NC, USA), and then ultrasonicated at 20,000 cycles/s for 1 min (Dr. Hielscher, Teltow, Germany). Homogenates were centrifuged at 4 °C and 10,000× *g* for 15 min, and the supernatants were collected. All the samples were stored at −85°C until analysis. Fructose concentrations in the plasma and the kidney homogenate were measured by using the colorimetric assay kit (BioVision Inc., Mountain View, CA, USA) according to the manufacturer’s instruction.

### 2.7. Statistical Analysis

The results are given as mean ± standard error of the mean (SEM); n is the number of rats. Gene and protein expression data were normalized to the mean of the control groups, which was arbitrarily set to one, and the relative changes were given as fold-changes over control. Student’s t-test was utilized to perform the statistical analyses for unpaired data, or the one-way ANOVA followed by the Bonferroni post hoc analysis was utilized where appropriate. Values were considered to be significantly different when the p-value was less than 0.05.

## 3. Results

### 3.1. The Influences of Dietary Fructose and the Supplementation of L. plantarum and L.helveticus on the Expression of Renal Insulin Signaling Effectors and Glucose Transporters 

The data with metabolic parameters, including body weight, caloric intake, the plasma level of glucose, insulin, and triglyceride, in rats upon fructose feeding, as well as *L. plantarum* and *L. helveticus* supplementations, have been published in our very recent study [26]. Briefly, in that paper, we have shown that intervention with dietary high-fructose or two probiotics did not change the body weight of rats. Dietary high-fructose-induced elevation in plasma glucose was not changed by both probiotics, but the increment in insulin level was diminished with *L. plantarum.* However, dietary fructose-induced increase in plasma triglyceride was reduced by the supplementation of *L. plantarum* or *L. helveticus* [26].

**Figure 1:**
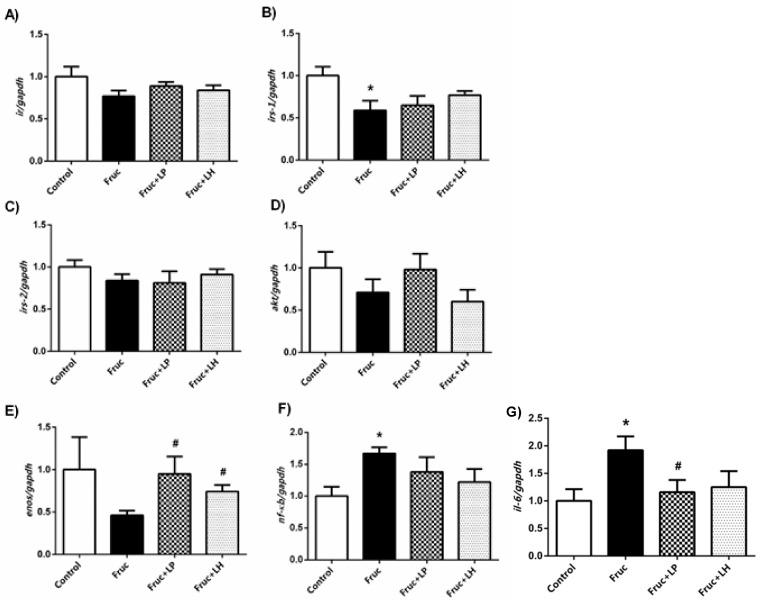
The mRNA expression levels of *ir* (**a**), *irs-1* (**b**), *irs-2* (**c**), *akt* (**d**), *enos* (**e**), *nf-ĸb* (**f**), and *il-6* (**g**) in kidney tissues of Control, Fruc, Fruc + LP, and Fruc + LH groups. Data were normalized using gapdh. Each bar represents the means from at least six rats. * *p* < 0.05, significantly different from the control; ^#^ *p* < 0.05, significantly different from fructose. Fruc: Fructose; LP: *L. Plantarum*; LH: *L. Helveticus*.

In the present study, we used the kidney tissue of the same animals, as used in the above paper, to investigate further biochemical and molecular aspects of nutrition with fructose and probiotics. Initially, we measured the expression level of renal insulin signaling elements: high-fructose consumption in rats reduced gene expression of *irs-1* but did not alter those of *ir* and *irs-2.* There was also a tendency toward reduction in both *akt* and *enos* mRNAs, but the differences did not achieve a significance level (Figure 1a–e). 

**Figure 2:**
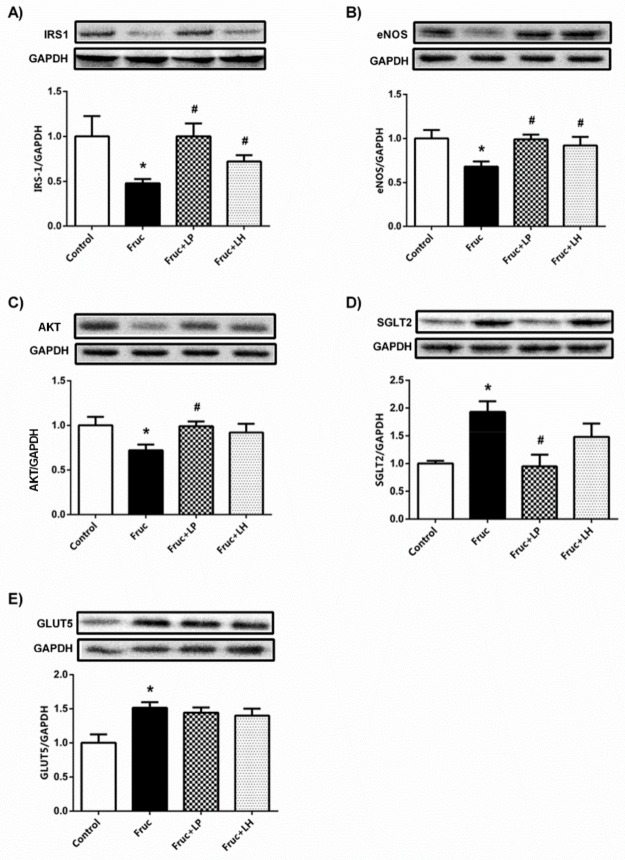
The protein expression levels of insulin receptor substrate (IRS-1) (**a**), protein kinase B (AKT) (**b**), endothelial nitric oxide synthase (eNOS) (**c**), sodium-glucose cotransporter-2 (SGLT2) (**d**), and glucose transporter type-5 (GLUT5) (**e**) in kidney tissues of Control, Fruc, Fruc + LP, and Fruc + LH groups. The protein levels were quantified using densitometry and normalized with GAPDH. Representative Western blot images are included above the corresponding figures. Each bar represents at least six rats. * *p* < 0.05, significantly different from control; ^#^ *p* < 0.05, significantly different from fructose. Fruc: Fructose; LP: *L. Plantarum*; LH: *L. Helveticus;* IRS-1: insulin receptor substrate 1; AKT: protein kinase B; eNOS: endothelial nitric oxide synthase; SGL2: sodium-glucose cotransporter-2.

However, probiotic supplementation with both *L. plantarum* and *L. helveticus* significantly increased *enos* mRNA expression in renal tissue of fructose-fed rats. Our Western blot results showed that high-fructose intake suppressed IRS-1, AKT, and eNOS protein expressions, which were significantly improved by *L. plantarum* and *L. helveticus* supplementations, except unchanged AKT expression in *L. helveticus* treated group (Figure 2a–c). Additionally, renal protein expressions of SGLT2 and GLUT5 were significantly augmented in fructose-fed rats. The supplementation of *L. plantarum* reduced the expression of SGLT2, but *L. helveticus* did not show any marked change. Moreover, both probiotics had no improving effect on the renal GLUT5 expression in fructose-fed rats (Figure 2d,e).

### 3.2. The Influences of Dietary Fructose and the Supplementation of L. plantarum and L. helveticus on Inflammatory Factors 

**Figure 3:**
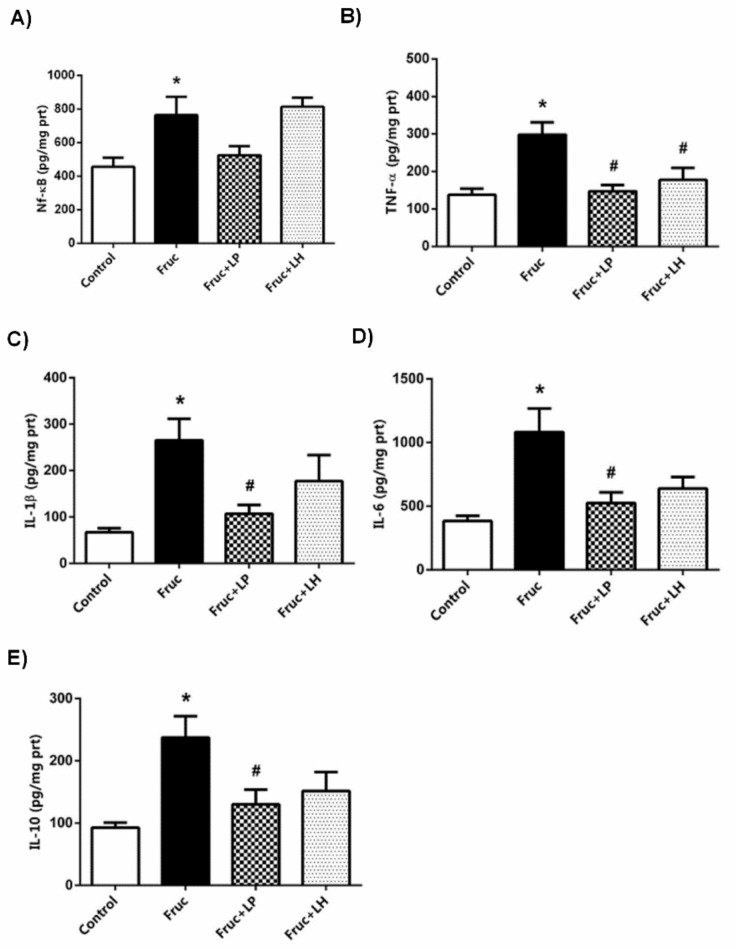
The levels of NF-ĸB (**a**), TNF-α (**b**), IL-1β (**c**), IL-6 (**d**), and IL-10 (**e**) in kidney tissues of Control, Fruc, Fruc + LP, and Fruc + LH groups. Each bar represents the means from at least six rats. * *p* < 0.05, significantly different from the control; ^#^ *p* < 0.05, significantly different from fructose. Fruc: Fructose; LP: *L. Plantarum*; LH: *L. Helveticus*; NF-ĸB: nuclear factor-kappa B; TNF-α: tumor necrosis factor α; IL: interleukin.

Dietary fructose elevated the renal tissue levels of NF-kB, TNF-α, IL-1β, IL-6, and IL-10. Besides, the expressions of NF-κB and IL-6 mRNAs were augmented. The supplementation of *L. plantarum* reduced the renal levels of TNF-α, IL-1β, IL-6, and IL-10, but not that of NF-κB, in high-fructose-fed rats. This probiotic supplementation also decreased the expression of IL-6 mRNA, but not that of NF-κB, in the kidney of high-fructose-fed rats. *L. helveticus* supplementation has a limited effect on the inflammatory parameters; thus, in consequence, this probiotic only decreased the renal level of TNF-α (Figure 1f,g and Figure 3a–e).

### 3.3. The Influences of Dietary Fructose and the Supplementation of L. plantarum and L. helveticus on Fructose Levels

**Figure 4:**
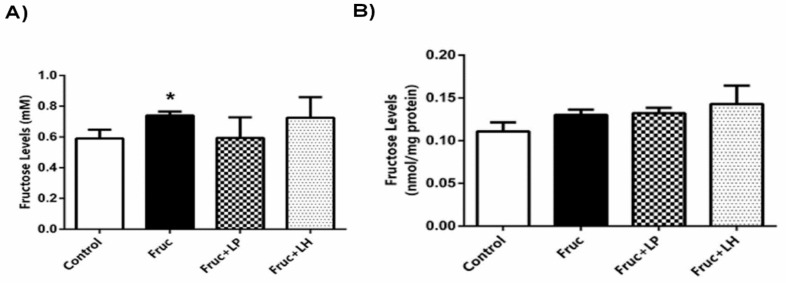
Plasma fructose concentration (**a**) and renal fructose content (**b**) in Control, Fruc, Fruc + LP, and Fruc + LH groups. Each bar represents at least six rats. * *p* < 0.05, significantly different from control. Fruc: Fructose; LP: *L. Plantarum*; LH: *L. Helveticus*.

The fructose intake of rats was calculated from liquid consumption and found to be 7.9 ± 0.2 g/kg body weight/day in fructose group; 7.7 ± 0.1 g/kg body weight/day in *L. plantarum* treated group; 7.7 ± 0.2 g/kg body weight/day in *L. helveticus* treated group. We also measured the fructose levels in plasma and kidney of rats: high-fructose consumption increased fructose concentration in the plasma but did not change in the kidney. Probiotic supplementation with *L. plantarum* or *L. helveticus* to high-fructose-fed rats did not affect the fructose concentration in the blood or kidney samples (Figure 4a,b).

## 4. Discussion

Although, some studies have reported the influence of several *Lactobacillus* species in high-fructose-induced metabolic syndrome [21,22,23,24,25,28], their mechanistic action in consumption of fructose, which is an essential part of the regular human diet, remains only partially understood. Recently, we have demonstrated the improvement of some metabolic parameters and renal protein expression of the antioxidant enzymes in fructose-fed rats with *L. plantarum* and *L. helveticus* [26]. In the current study, results revealed that *L. plantarum* supplementation has alleviating effects on the suppression of insulin signaling elements, induction of inflammatory markers, and upregulation of SGLT2 in renal tissue of fructose-fed rats. This may offer a novel molecular understanding as well as an alternative approach to excess fructose consumption.

Regarding basic knowledge, it is well established that insulin effectively controls glucose homeostasis and lipid metabolism. The action of insulin in the cells is initiated by the activation of its receptor (IR), and the signal through IRS-1 and IRS-2 transmits to its main effectors AKT and eNOS. Previously, we showed that high-fructose intake altered the expression levels of insulin signaling effectors, IR, IRS-1, AKT, and eNOS in several tissues, including blood vessels, adipose tissue, and liver of rats [3,4,5,6,7]. In studies with kidney tissue of high-fructose-fed rats, reduced expression or phosphorylation of IR, IRS-1, and AKT [9,10] were consistent with our present findings in which we demonstrated a downregulation in renal protein expression of IRS-1, AKT, and eNOS, as well as in their gene expression pattern to some extent, but not thoroughly. These results indicate that renal insulin signaling effectors are downregulated, particularly at the posttranslational level, in the consumption of high-fructose, revealing a possible insulin resistance condition in the kidney. The above studies also proposed that there was a possible connection between inflammation and impaired insulin signaling at the organ level, including blood vessels, adipose, liver or kidney tissues, as mentioned in the introduction of this paper. This proposal was supported by the current findings, in which we determined that dietary high-fructose increased the renal tissue levels of NF-κB, TNFα, IL-1β, IL-6, and IL-10, as well as the expression of NF-κB and IL-6 mRNAs. On the other hand, the reduced expression of insulin receptor and IRS-1 of the kidney cortex was proposed to contribute to hyperglycemia in high-fat-diet-fed mice or type 2 diabetic rats [29,30]. The kidneys play an essential role in glycemic control through renal gluconeogenesis and tubular glucose reabsorption. The glucose about 90% was reabsorbed via SGLT2 transporter in the proximal tubule [31]. The renal expression of SGLT2 was increased in hyperglycemic condition with a high-fructose diet [14]. In this study, upregulation of specific glucose transporter SGLT2 suggests that tubular glucose reabsorption is raised in high-fructose-fed rats as evidenced by high levels of plasma glucose and insulin. This finding also indicates a relationship between renal insulin signaling and SGLT2 expression in the insulin resistance condition, as previously suggested [32]. Besides, the expression of GLUT5, which is a specific fructose transporter, was found to be increased in renal tissue of rat or mice fed with a high-fructose diet [12,13]. Deletion of GLUT5 caused a marked reduction in fructose absorption in mice fed with high-fructose [33]. Herein, a noticeable enhancement in renal GLUT5 protein expression suggests that fructose concentration might be increased in blood and kidney of rats. We determined an elevation in the concentration of fructose in the blood as well as a trend to increase in the kidney. It was recently shown that low-dose dietary fructose is converted to glucose and organic acids in the small intestine, but high-dose fructose overcomes the intestinal capacity and spills over to the liver and blood, as well as its some part stays in the colonic lumen [34,35]. All these indicate the availability of a high amount of fructose in the systemic circulation and its exposure to the organs.

Several *Lactobacillus* species alone or in combination, including *L. acidophilus*, *L. casei, L. curvatus, L. plantarum, L. reuteri, L. kefiri,* and *L. rhamnosus,* were demonstrated to produce functional effects in high-fructose-induced metabolic disturbances via the reduction in oxidative stress, lipogenic genes expression, or pro-inflammatory cytokines [21,22,23,24,25,28]. In our very recent study, we noticed an improving effect of *L. plantarum* and *L. helveticus* on dietary fructose-induced metabolic changes with hyperinsulinemia and hypertriglyceridemia, as well as increased creatinine and urea. These may also involve a possible influence on renal insulin effectors. In this study, supplementation of *L. plantarum* in high-fructose-fed rats promoted the renal protein expression of IRS-1, eNOS, and AKT more effectively than did *L. helveticus.* Additionally, both probiotics exerted an improving effect on eNOS mRNA expression. In this sound, oral administration of *L. plantarum* Ln4 strain (Ln4) reduced weight gain and plasma triglyceride level and insulin resistance index associated with increases in hepatic gene expression of *irs2* and *akt2* in mice fed with a high-fat diet [36]. Although herein, we showed no change in the expression of *ir* and *irs-2* mRNAs with dietary fructose or probiotics, the insulin signaling pathway is on the target of these dietary interventions, but the actions may alter at post-receptor levels in the different tissues. The production and expression of cytokines, which are the essential determinant of inflammatory status, was shown to be reduced by the treatment with *L. plantarum, L. reuteri, L. rhamnosus, or L. kefiri* in blood, adipose tissue, and liver of rodents fed on a high-fructose diet [23,24,25,28]. These data are consistent with our current findings that the renal tissue levels of TNFα, IL-1β, IL-6, and IL-10, as well as expression of IL-6 mRNA, were suppressed with *L. plantarum* supplementation in high-fructose-fed rats. This attenuation in cytokines production may also contribute to the substantial improvement in renal insulin signaling. Likewise, administration of heat-killed *L. plantarum* L-137 (HK L-137) was found to inhibit inflammation and improve insulin signaling in both visceral and subcutaneous adipose tissue in obese rats via decreasing expression of cytokines and increasing phosphorylation of AKT, respectively [37]. Also, dead *L*. *plantarum* treatment decreased the expression of iNOS and IL-1β in intestine or liver of streptozotocin-induced diabetic mice [38]. Given the probiotics may have beneficial effects, at least, by suppressing inflammatory factors, the hyperglycemic condition-induced destruction may overcome. To our knowledge, no study has investigated the effect of the probiotics on the glucose transporters SGLT2 and GLUT5, which are the major glucose and specific fructose transporters, respectively, in the kidney. Importantly, our data with glucose transporters showed that the treatment with *L. plantarum*, but not *L. helveticus*, impaired increased renal protein expression level of SGLT2 in high-fructose-fed rats, indicating that former exerts an effort to limit glucose reabsorption. Incompatible with this statement, in our related study, blood insulin level was lower in the *L. plantarum* treatment group than the *L. helveticus* group, representing a little requirement of insulin to equilibrate the blood glucose, despite the fact that glucose levels were still high in both treatment groups [26]. In contrast, for GLUT5, no such improvement was observed. Accordingly, fructose concentration in blood or kidney of rats was not impaired following the probiotics treatment. All these simply indicate that there is an exertion to restrict tubular reabsorption of glucose, but not fructose, by supplementation of *L. plantarum*.

## 5. Conclusions

In conclusion, improvement in the renal insulin signaling pathway, SGLT2 expression, and inflammatory reactivity by *L. plantarum* supplementation may provide a novel molecular explanation, as well as therapeutic approach, for the renal dysregulation induced by dietary high-fructose.

## Figures and Tables

**Table 1:** Primer sequences of *ir, irs-1, irs-2, akt, enos, nf-κb, il-6,* and internal standard *gapdh* used for the mRNA expression determination of qRT-PCR.

Gene	Forward Primer Sequence (5′→3′)	Reverse Primer Sequence (3′→5′)
*ir*	GTGCTGCTCATGTCCTTAGA	AATGGTCTGTGCTCTTCGTG
*irs-1*	GCCAATCTTCATCCAGTTGC	CATCGTGAAGAAGGCATAGG
*irs-2*	CTACCCACTGAGCCCAAGAG	CCAGGGATGAAGCAGGACTA
*akt*	GAAGAAGAGCTCGCCTCCAT	GAAGGAGAAGGCCACAGGTC
*enos*	TGCACCCTTCCGGGGATTCT	GGATCCCTGGAAAAGGCGGT
*nf-κb*	GGGTCAGAGGCCAATAGAGA	CCTAGCTTTCTCTGAACTGCAAA
*il-6*	CCAGTTGCCTTCTTGGGACT	GCCATTGCACAACTCTTTTCTCA
*gapdh*	TCCTTGGAGGCCATGTGGGCCAT	TGATGACATCAAGAAGGTGGTGAAG
